# A Hybrid Chalcone Combining the Trimethoxyphenyl and Isatinyl Groups Targets Multiple Oncogenic Proteins and Pathways in Hepatocellular Carcinoma Cells

**DOI:** 10.1371/journal.pone.0161025

**Published:** 2016-08-15

**Authors:** Lili Cao, Lijun Zhang, Xiang Zhao, Ye Zhang

**Affiliations:** 1 Department of Cell Biology, School of Basic Medical Sciences, Peking University, Beijing, China; 2 School of Life Sciences, Peking University, Beijing, China; University of Pittsburgh, UNITED STATES

## Abstract

Small molecule inhibitors that can simultaneously inhibit multiple oncogenic proteins in essential pathways are promising therapeutic chemicals for hepatocellular carcinoma (HCC). To combine the anticancer effects of combretastatins, chalcones and isatins, we synthesized a novel hybrid molecule 3’,4’,5’-trimethoxy-5-chloro-isatinylchalcone (3MCIC). 3MCIC inhibited proliferation of cultured HepG2 cells, causing rounding-up of the cells and massive vacuole accumulation in the cytoplasm. Paxillin and focal adhesion plaques were downregulated by 3MCIC. Surprisingly, unlike the microtubule (MT)-targeting agent CA-4 that inhibits tubulin polymerization, 3MCIC stabilized tubulin polymers both in living cells and in cell lysates. 3MCIC treatment reduced cyclin B1, CDK1, p-CDK1/2, and Rb, but increased p53 and p21. Moreover, 3MCIC caused GSK3β degradation by promoting GSK3β-Ser9 phosphorylation. Nevertheless, 3MCIC inhibited the Wnt/β-catenin pathway by downregulating β-catenin, c-Myc, cyclin D1 and E2F1. 3MCIC treatment not only activated the caspase-3-dependent apoptotic pathway, but also caused massive autophagy evidenced by rapid and drastic changes of LC3 and p62. 3MCIC also promoted cleavage and maturation of the lysosomal protease cathepsin D. Using ligand-affinity chromatography (LAC), target proteins captured onto the Sephacryl S1000-C_12_-3MCIC resins were isolated and analyzed by mass spectrometry (MS). Some of the LAC-MS identified targets, i.e., septin-2, vimentin, pan-cytokeratin, nucleolin, EF1α1/2, EBP1 (PA2G4), cyclin B1 and GSK3β, were further detected by Western blotting. Moreover, both septin-2 and HIF-1α decreased drastically in 3MCIC-treated HepG2 cells. Our data suggest that 3MCIC is a promising anticancer lead compound with novel targeting mechanisms, and also demonstrate the efficiency of LAC-MS based target identification in anticancer drug development.

## Introduction

Hepatocellular carcinoma (HCC) is a life-threatening malignant disease with nearly 782,500 new cases and 745,500 deaths around the world every year [1]. Although hepatic resection is the priority treatment for early-stage HCC, the reported 5-year disease-free survival rate after resection was 53.5% if more advanced patients reserved for liver transplantation were excluded, but was only 31.6% if liver transplantation was not licensed in hospitals [2]. Despite a survival rate improvement, liver transplantation is seriously limited by availabilities of donor livers and qualified hospitals. For those with conditions not treatable by surgery or ablation, systemic chemotherapy is disappointing [3,4]. None of the drugs effective for other types of cancers had been proved to have any benefit for HCC patients, until the success of the phase III SHARP trial for sorafenib, which led to a new era of targeted therapy for HCC [5]. Encouraged by the SHARP trial, more than fifty small molecules and mAbs have been under clinical evaluation [6,7]. However, up to now most of the terminated trials were either ineffective or inferior to sorafenib.

Dozens of negative trials in HCC chemotherapy reflect our lack of deep understanding of the molecular biology of HCC and effective combinations of target hitting in molecular therapy [8–12]. Recent studies in HCC have uncovered multiple mutational or regulatory disorders that are mainly clustered in the p53/cell-cycle control, telomere/chromatin remodeling, and signal transduction including the Wnt/β-catenin, Ras-MAPK, AKT-PI3K-mTOR, notch, JAK/STAT, and hypoxia-angiogenesis pathways [13–20]. In principle, those multitarget drugs with their target signatures match the molecular signature of disorders in HCC should display most powerful anticancer activities, which may explain why sorafenib is superior to dozens of other drugs. Since sorafenib’s well-known targets are VEGFR, PDGFR and RAF, it had been considered as a kinase inhibitor mainly targeting angiogenesis [20,21]. However, more detailed study reveals that sorafenib’s targets are extremely plenty, i.e., it binds to at least 11 targets with affinities higher than or similar to VEGFR (Kd ~ 60 nM), and also binds to another 13 targets comparable to RAF1 and BRAF(V600E) (Kd 230~260 nM) [22]. Notably, these 24 targets in a sum are highly correlated with pathway disorders in HCC.

**Fig 1:**
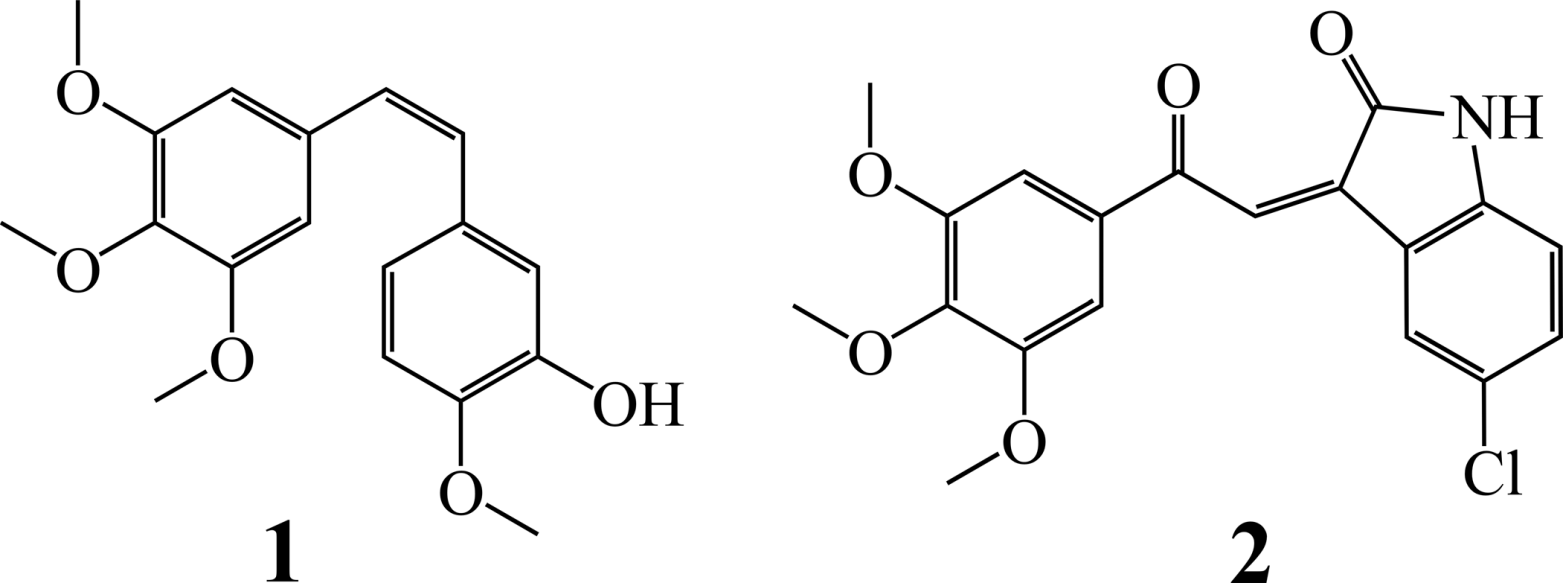
Chemical structures. **1**. Combretastatin A-4 (CA-4). **2**. ‘,4’,5’-trimethoxy-5-chloro-isatinylchalcone (3MCIC).

In spite of the progress in searching kinase inhibitors, more efforts are needed in exploring targetable molecules in the cytoskeleton system of HCC cells. Microtubule (MT)-targeting agents such as taxols, colchicines and vinblastines are the most powerful and widely used anticancer compounds [23]. In contrast to taxols, which stabilize tubulin polymers, colchicines and vinblastines inhibit tubulin polymerization. Recently, the anti-MT agent combretastatin A-4 (CA-4, Fig 1 compound **1**), a natural-occurring *cis*-stilbene that shares the 3,4,5-trimethoxyphenyl pharmacophore with colchicine, has aroused significant interests, since it not only has potent cytotoxic effect resulting from MT collapse, but also disrupts blood vessels in cancer tissues [24,25]. On the other hand, natural-occurring and synthetic chalcones with potent anticancer activities have been documented progressively [26,27]. It is believed that chimeric compounds bearing two or more bioactive fragments can enhance or fine-tune their anticancer effects [28,29]. Thus, synthetic chalcones containing the trimethoxyphenyl fragment have showed promising anticancer activities [30,31]. Similarly, a hybrid multitarget molecule that combines the structures of anti-MT agents and VEGFR2 inhibitors has potent anticancer effects across the entire NCI-60 tumor cell panel [32].

In this paper, we report the anticancer effects, mechanism and target identification of a novel chimeric compound (*Z*)-5-chloro-3-(2-(3,4,5-trimethoxyphenyl)-2-oxoethylidene)indolin-2-one or 3’,4’,5’-trimethoxy-5-chloro-isatinylchalcone (3MCIC) (Fig 1 compound **2**), which contains trimethoxyphenyl, chalcone and isatinyl moieties, and is more potent than sorafenib in killing the cultured liver cancer HepG2 cells. Isatins are a class of natural-occurring and synthetic substances that have wide ranges of biological and pharmacological activities [33,34]. For examples, certain isatin derivatives block cell cycle and induce cell death by inhibiting CDK2 and glycogen synthase kinase 3β (GSK3β) [35]. Derivatives of 5,7-dibromoisatin have potent anticancer activities due to dual inhibition of both tubulin polymerization and AKT activation [36]. Hybrid anticancer compounds prepared from isatin and podophyllotoxin were documented [37]. However, hybrid molecules linking the trimethoxyphenyl and isatinyl groups in the form of chalcones were not reported.

Our data showed that 3MCIC caused drastic morphological changes in the HepG2 cells, accompanied with multiple alternations of the cytoskeleton system. 3MCIC affected the cell-cycle regulation network at multiple levels, and inhibited the Wnt/β-catenin-GSK3β pathway. Both the autophagic and apoptotic pathways were activated upon 3MCIC treatment. To identify 3MCIC’s targets, we prepared a ligand-affinity chromatography (LAC) column by coupling 3MCIC to the Sephacryl S1000 resins. Target proteins obtained by LAC were analyzed with SDS-PAGE and mass spectrography (MS). The results revealed that 3MCIC bound to multiple proteins including septins, the fourth component of the cytoskeleton. Our work suggest that 3MCIC is a promising anticancer lead compound with novel targeting mechanisms, and also demonstrate the efficiency of LAC-MS based target identification in anticancer drug development.

## Materials and Methods

### Reagents

Rabbit polyclonal antibodies for phosphorylated p-CDK1/2 (Thr14Tyr15), CDK2, CDK4, p-Rb (Ser567P), EF-1α1/2, EBP1, septin-2; and mouse monoclonal antibodies for cyclin A (E23.1), cyclin E (HE12), CDK1/Cdc2 (17), E2F1 (KH95), c-Myc (9E10), pan-cytokeratin (PK110), caspase-3 (E-8), HIF-1α (H1α 67) were purchased from Santa Cruz Biotechnology (Santa Cruz, CA). Rabbit monoclonal antibodies for paxillin (Y113), vimentin (SP20), PARP (46D11), p-GSK3β (Ser9) (5B3), LC3A/B (D3U4C), p62/SOSTM1 (D10E10); and mouse monoclonal antibody for Rb (4H1) were from Cell Signaling Technology (Beverly, MA). Mouse monoclonal antibodies for calnexin (37) and cathepsin D (C5) were from BD Biosciences (San Joes, CA) and GenWay Biotech (San Diego, CA), respectively. Rat monoclonal antibody for GSK3β (272536) was from R & D systems (Minneapolis, MN). Mouse monoclonal antibody for α-tubulin (DM1A) was from Sigma-Aldrich (St. Louis, MO). Rabbit monoclonal antibody for cyclin D1 and mouse monoclonal antibody for cyclin B1 (GNS11) were from Thermo Fisher Scientific (Fremont, CA). Mouse monoclonal antibodies for p21 (DSC-60) and p53 (DO-1) were from MBL (Naka-ku Nagoya, Japan). Rabbit monoclonal antibody for activated caspase-3 (E83-77) was from Abcam (Cambridge, UK).

### Cell culture

Human liver cancer cell line HepG2, breast cancer cell lines MDA-MB-231 and MCF-7, nasopharyngal carcinoma cell line CNE, colon cancer cell line HCT-116, and human fetal liver cell line L02 were obtained from the Cell Bank of the Committee on Type Culture Collection, Chinese Academy of Sciences (Shanghai, China). Cells were grown at 37°C in the RPMI-1640 or DMEM medium (Gibco) supplemented with 10% fetal bovine serum (FBS) and in a humidified atmosphere supplemented with 5% CO_2_. Exponentially growing cells were used for all experiments.

### Cell viability assay

The acid phosphatase assay was used to evaluate cell viability [38]. Cells were seeded in 96-well plates at densities of 3,500–5,500 cells per well, incubated for 24 h and exposed to various concentrations of 3MCIC. DMSO was used as the solvent control. After another 48 h incubation, the plates were washed with 200 μl phosphate-buffered saline (PBS). Added to each well was 100 μl assay buffer containing 0.1 M sodium acetate (pH 5.0), 0.1% Triton X-100, and 5 mM p-nitrophenyl phosphate. The plates were incubated at 37°C for 2 h. The reaction was stopped by adding 30 μl of 1 M NaOH, which caused color development instantly. Absorbance at 405 nm was recorded using a microplate reader (GmbH5082, TECAN). Dose-response curves and the 50% growth inhibition concentration (IC_50_) values were obtained by the GraphPad Prism software.

### Immunofluorescence

Procedures were following our previous works [39–41]. Briefly, HepG2 cells were plated and grew on coverslips, treated with 3MCIC for indicated times, washed with pre-warmed PBS, and fixed with 4% paraformaldehyde for 10 min. The cells were permeated with 0.5% Triton-100 for 5 min, blocked with 3% BSA for 40 min, washed, and incubated with primary antibodies for 1 h at room temperature. After washing, cells were stained with Alexa Fluor 488-conjugated secondary antibodies (Invitrogen) for 1 h. F-actins were stained with Alexa Red-phalloidin (Sigma-Aldrich). After a brief exposure to Hoechst 33342, the coverslips were immersed with the antifade mounting medium containing p-phenylenediamine (Sigma-Aldrich), sealed and observed under a fluorescence microscope (DM5000B, Leica).

### Western blotting

Cells grown at a density of 1×10^6^/dish were incubated with 3MCIC, washed with PBS and lysed by incubation on ice for 30 min with a buffer containing 50 mM Tris-HCl (pH 8.0), 150 mM NaCl, 0.1% SDS, 1% Nonidet-P40, 1% Triton X-100, 5 mM EDTA, 1 mM phenylmethylsulfonyl fluotide (PMSF) and the protease inhibitor cocktail (Roche). Phosphatase inhibitors were added when analysis of phosphorylated proteins was needed. The cell lysates were centrifuged at 14,000×*g* for 10 min, and the supernatants were collected. Protein concentrations of the lysates were assayed by the Bradford method. For SDS-PAGE, cell lysates were boiled in the reducing sample buffer, and equal amount of proteins (40 μg) were loaded onto the gels. After electrophoresis, proteins were transferred to a nitrocellulose membrane (Millipore). The membrane was blocked, and incubated sequentially with primary and HRP-coupled secondary antibodies. Calnexin was used as the sample loading control. Protein bands were visualized by the ECL technique (Pierce), scanned, and quantified by the Quantity One software.

### Analysis of polymerized and soluble tubulins

Separation of insoluble polymerized-tubulin (P-tubulin) in MTs from soluble tubulin (S-tubulin) dimers was performed following previous reports [42,43]: To analyze drug-induced MT changes in living cells [42], HepG2 cells were cultured at 37°C with FBS-supplemented RPMI-1640 media containing 3MCIC, paclitaxel, or colchicine for 6 or 12 h, respectively. Then cells were lysed at 25°C with agitations for 10 min with a hypotonic lysing buffer (pH 6.8) containing 20 mM Tris-HCl, 1 mM MgCl_2_, 2 mM EGTA, 0.5% NP40, 2 mM PMSF, and the protease inhibitor cocktail (Roche). The lysates were centrifuged at 15,000×*g* for 10 min at 4°C. The pellets were suspended in the reducing SDS-PAGE sample loading buffer and boiled for 10 min to dissolve P-tubulin. Supernatants containing S-tubulin were treated identically. Samples from the supernatant and the pellet were adjusted to equal volumes prior to gel loading for electrophoresis. Tubulin contents were assayed by Western blotting. To analyze drug-induced MT changes in cell lysates [43], untreated HepG2 cells were lysed at 25°C for 10 or 30 min in 200 μl of the above hypotonic lysing buffer containing 3MCIC or paclitaxel, respectively. P- and S-tubulins were separated and detected as above.

### TUNEL assay for apoptosis

Terminal deoxynucleotidyl transferase-mediated dUTP nick end labeling (TUNEL) was performed according to our previous report [41]. Briefly, cells grown on coverslips were treated with 3MCIC or vehicle for 12 h, fixed with 4% paraformaldehyde, and then permeabilized with 0.1% Triton X-100. DNA breaks in apoptotic cells were labeled with the TUNEL BrightRed Apoptosis Detection Kit (Vazyme Biotech Co., Nanjing, China), and observed under a fluorescence microscope.

### Target identification by ligand-affinity chromatography-mass spectrometry (LAC-MS)

3MCIC was coupled to the Sephacryl S1000 resins by our group [44] using a 12 carbon-spacer to facilitate access of target proteins (S1 Fig). About 0.8 ml of the 3MCIC-resins was packed into a LAC column. Untreated HepG2 cells were lysed on ice-bath with a hypotonic buffer containing 50 mM Tris-HCl (pH 8.0), 30 mM NaCl, 1% NP40. The lysates were centrifuged at 14,000×*g* for 10 min at 4°C. Supernatants were collected, diluted 1:2.5 (V:V) with water, and then loaded onto the LAC column which was pre-equilibrated at 4°C with the column buffer (50 mM Tris-HCl, pH 7.4, 30 mM NaCl, 0.04% NP40). The column was washed with the column buffer until no proteins were detected in the flow-through fractions by the Bradford method. Target proteins captured onto the column were eluted with a high salt buffer containing 50 mM Tris-HCl (pH 7.4), 0.2 M NaCl, and 0.04% NP40. Peak fractions in the eluents were detected by the Bradford method and anaylzed with SDS-PAGE. After staining with Coomassie brilliant blue R-250, a major and a minor bands on the PAGE gel were visualized, excised, digested with trypsin, and subjected to MS (LTQ Orbitrap Velos Pro, Thermo). Target proteins in the LAC eluents were also identified by Western blotting.

### Statistical Analysis

Data were presented as means ± SEM (standard error of mean). Statistical analysis was performed using the Student’s *t*-test. Statistical significance was defined as p<0.05.

## Results

### HepG2 cells are sensitive to 3MCIC

**Fig 2:**
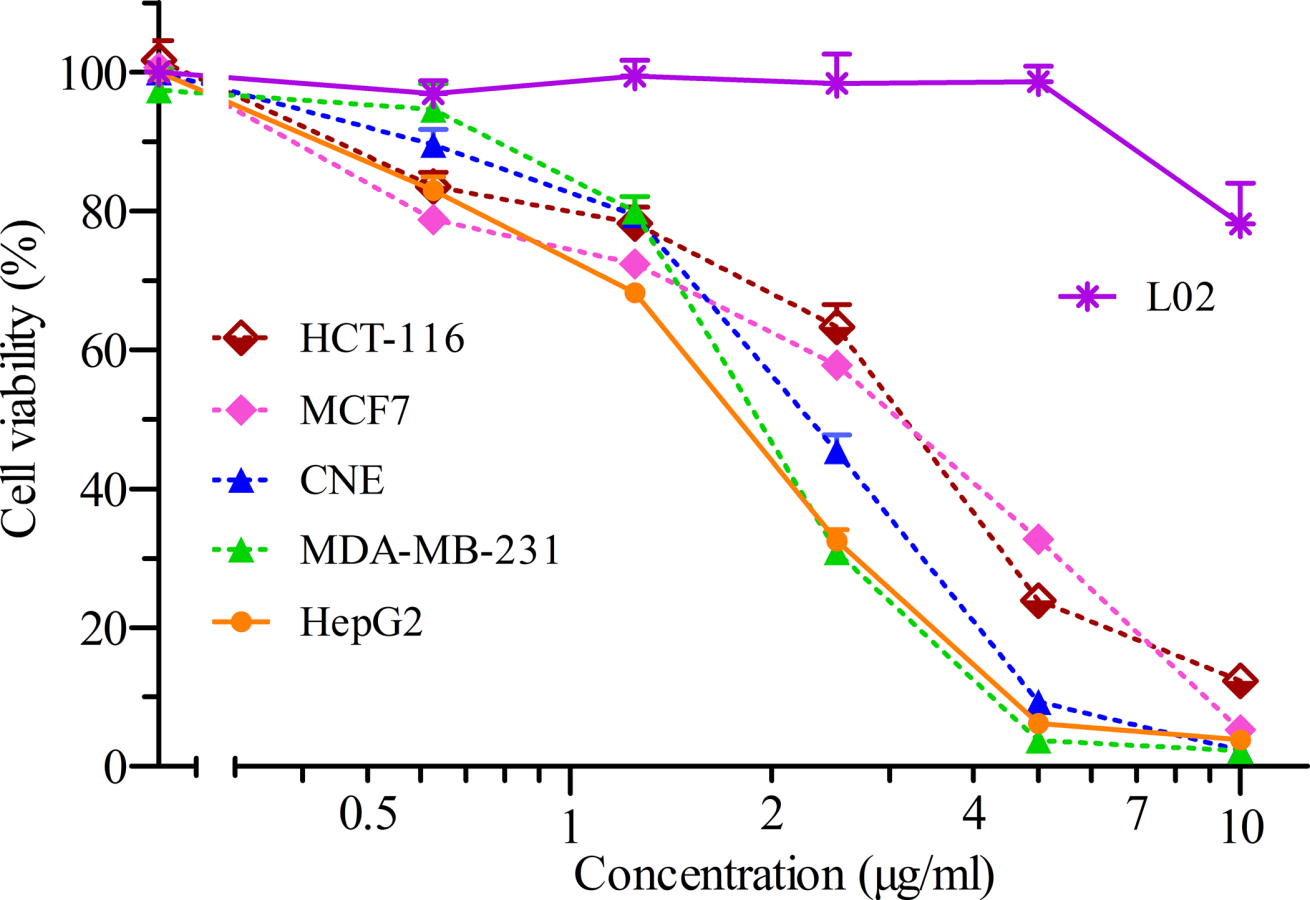
Cell viability assay. Dose-response curves of five cancer cell lines and the fetal liver L02 cells treated with different concentrations of 3MCIC for 48 h. The bars are means ± SEM (n = 6 or 8).

We analyzed the growth inhibitory effect of 3MCIC against a panel of five human cancer cell lines (Fig 2). The IC_50_ for HepG2 was 1.7 μg/ml (4.5 μM), which was the lowermost value among the tested cells. The IC_50_ (μg/ml) for other cells were: 1.9 for MDA-MB-231, 2.2 for CNE, 2.6 for MCF-7, and 2.9 for HCT-116, respectively. Contrarily, only slight inhibition of the fetal liver cell line L02 was observed under the tested 3MCIC dosages, indicating that 3MCIC had selective cytotoxicity on liver cancer cells over fetal liver cells. We also found that 3MCIC was much more powerful than sorafenib in inhibiting and killing cultured HepG2 cells, since under the same assay settings, sorafenib's IC_50_ for HepG2 was 20.1 μM.

### 3MCIC induces rounding and accumulation of vacuoles in cultured HepG2 cells

**Fig 3:**
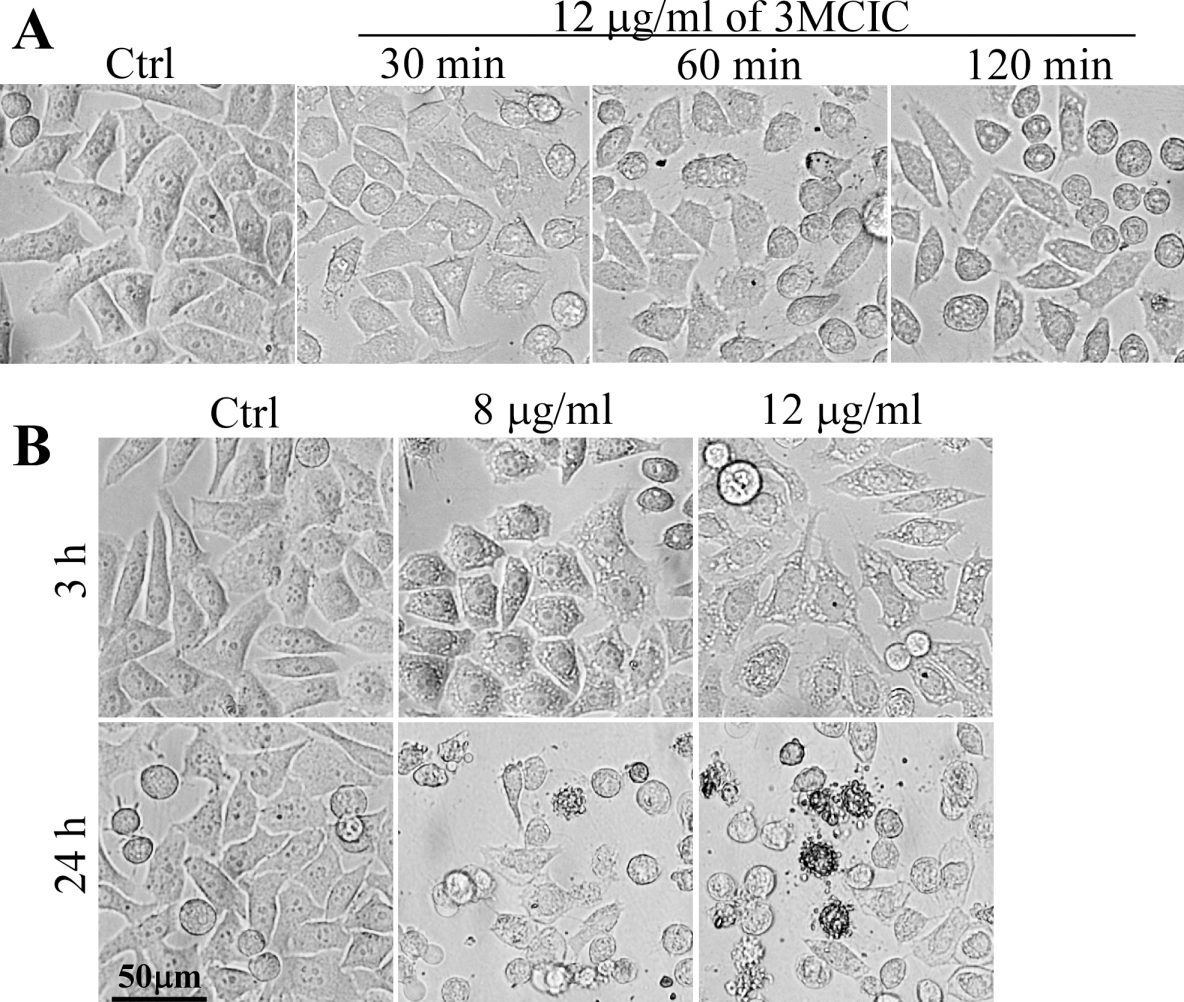
Morphological changes in 3MCIC-treated HepG2 cells. **(A)** HepG2 cells in 6-well plates were incubated with 12 μg/ml 3MCIC for 30, 60 and 120 min, and observed under a phase-contrast inverted microscope. **(B)** Prolonged incubation of HepG2 cells with 8 and 12 μg/ml 3MCIC for 3 and 24 h, respectively.

Treating HepG2 cells with 12 μg/ml of 3MCIC caused retraction and rounding of the cells within 1 h. Moreover, extensive filopodia protruded from the cell surfaces and multiple vacuoles developed in the cytoplasm (Fig 3A). The vacuoles enlarged dramatically after prolonged treatment with 8 or 12 μg/ml of 3MCIC for 3 h (Fig 3B). After 24 h incubation with 3MCIC, numerous round cells displayed multiple apoptotic body-like blebs on the cell surfaces. However, the phenotypes were heterogenic, since some of the cells remained spread, and their cytoplasmic vacuoles were fused into a single huge vacuole (Fig 3B), suggesting that complex and non-classical forms of cell death such as parapotosis might occurred [45].

### 3MCIC diminishes paxillin and focal adhesion plaques in HepG2 cells

**Fig 4:**
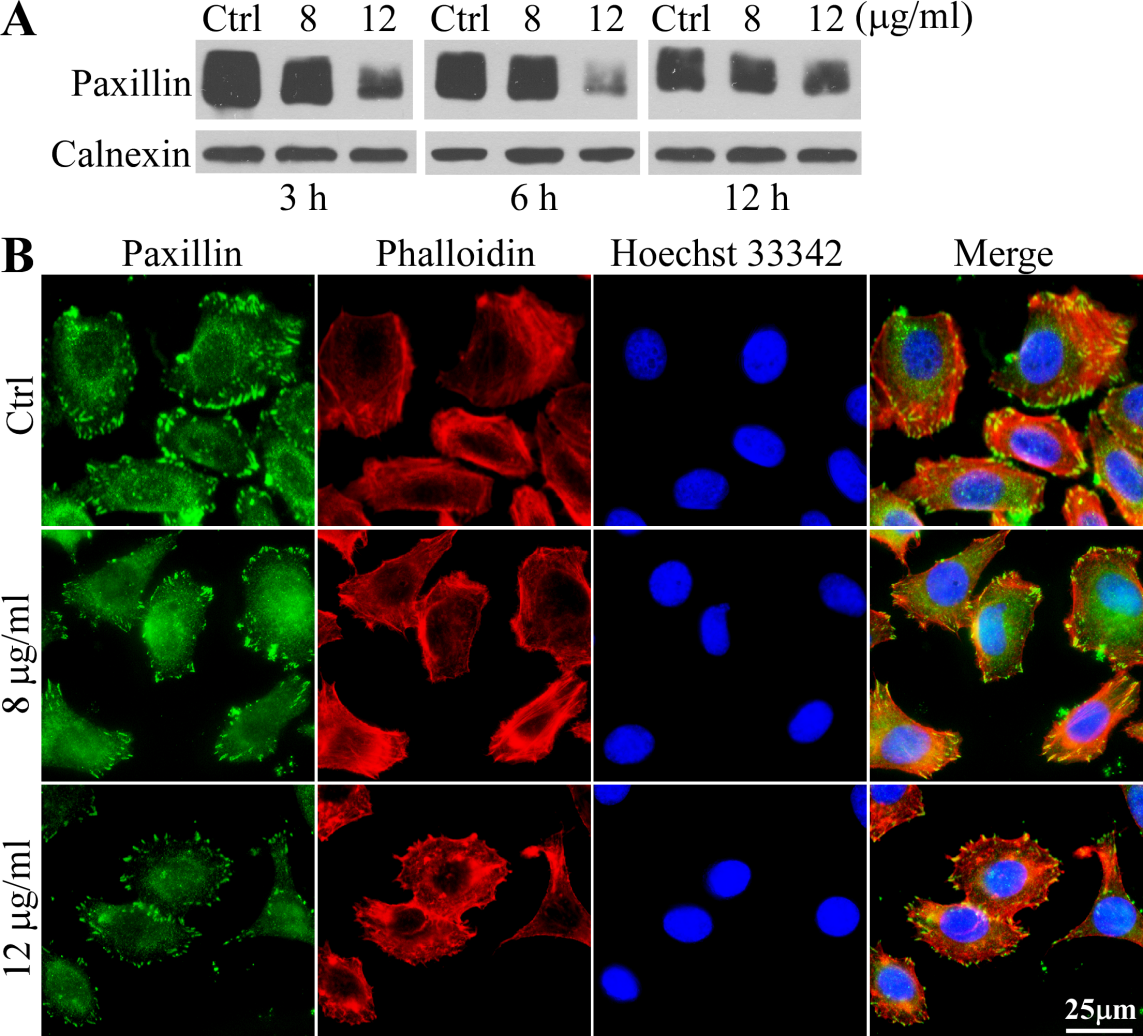
Diminish of paxillin and focal adhesion plaques in 3MCIC-treated HepG2 cells. **(A)** Cells were treated with 3MCIC and assayed by Western blotting. Calnexin was used as loading control. **(B)** Immunofluorescence of HepG2 cells stained with paxillin mAb (green), phalloidin (red) and Hoechst 33342, after incubation with 8 or 12 μg/ml 3MCIC for 3 h.

To investigate the mechanism underlying the rounding phenotype in 3MCIC-treated cells, we analyzed paxillin contents by Western blotting. The paxillin bands were diffused, due to heterogeneity of phosphorylation (Fig 4A). The results indicated that paxillin levels were significantly reduced by 3MCIC in a dose-dependent way. Paxillin can be used as a marker of the focal adhesion plaque in the immunofluorescent assay. In the control cells, adhesion plaques densely aligned on the edges of lamellipodia, where the F-actin stress fibers attached to the adhesion plaques regularly. However, 3MCIC treatment for 3 h dramatically decreased the number, size and brightness of adhesion plaques. Moreover, F-actin fibers became entangled, losing the parallel arrangement of stress fibers (Fig 4B).

### Polymerized tubulin is increased by 3MCIC treatment

**Fig 5:**
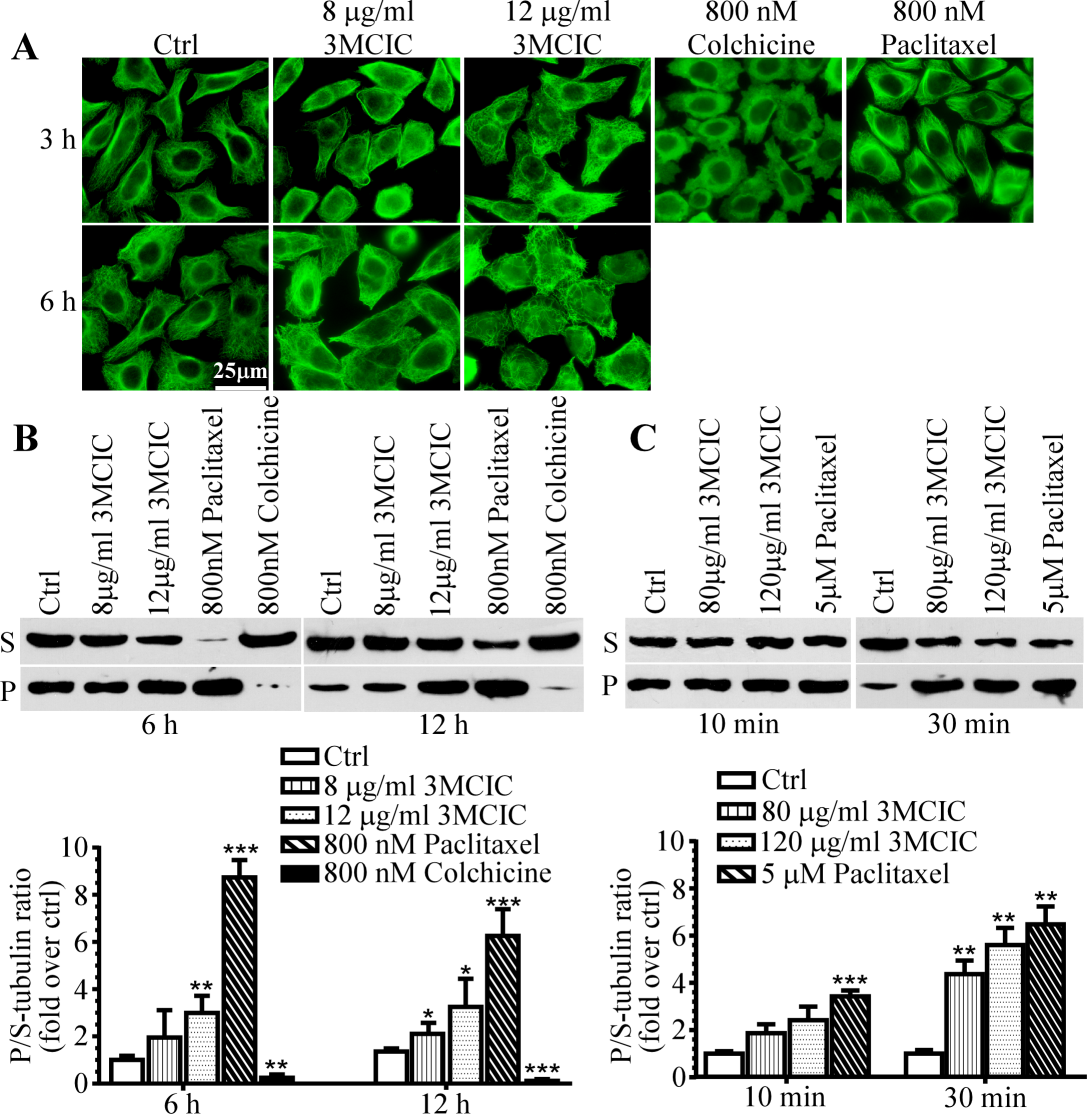
Influence of tubulin polymerization by 3MCIC. HepG2 cells were incubated at 37°C with 3MCIC, paclitaxel or colchicine, and assayed with α-tubulin mAb by immunofluorescence **(A)** and Western blotting **(B)** after separation of soluble (supernatant, S) and polymerized (pellet, P) tubulins. **(C)** HepG2 cell lysates were incubated at 25°C for 10 or 30 min with 3MCIC or paclitaxel respectively, and analyzed by Western blotting. In both **B** & **C**, normalized changes of the band-intensity ratios of P/S-tubulin in drug-treated groups over controls were plotted. *P<0.05, **P<0.01 and ***P<0.001 vs control (n = 3).

We synthesized 3MCIC as an analog of the anti-MT drug CA-4. The effect of 3MCIC on MT assembly was studied. MT stabilizer paclitaxel and polymerization inhibitor colchicine were used as controls and their effects were confirmed (Fig 5). Unexpectedly, 3MCIC treatment significantly increased P-tubulin in cultured HepG2 cells in a dose-dependent way. Although MT immunofluorescence increased in 3MCIC-treated cells (Fig 5A), the regular arrangement of MTs disappeared, replaced instead with entangled mashes and aggregated patches, especially under higher dosage and prolonged treatment. To confirm the immunofluorescence results, we separated P-tubulin (pellet) and S-tubulin (supernatant) in cell lysates by centrifugation, and then quantified the normalized ratio of P/S-tubulin by Western blotting (Fig 5B). Statistical analysis revealed that 3MIC increased the P/S-tubulin ratio in cultured HepG2 cells in a way similar with paclitaxel, but opposite to colchicine. Incubation of the HepG2 cell lysates with 3MCIC at 25°C for 10 or 30 min also increased the P/S-tubulin ratio (Fig 5C). Note that the same lysis-incubation buffer was used for living cells (Fig 5B) and for cell lysates (Fig 5C). In such a buffer, MTs in the control samples were gradually disassembled into S-tubulin upon incubation, but tubulin polymerization could not proceed. Addition of either 3MCIC or paclitaxel to the buffer retarded MT disassembling. Therefore, the P/S-tubulin ratios in either 3MCIC- or paclitaxel-treated samples were higher than that in control. However, for both 3MCIC and paclitaxel, the dosages required for blocking MT disassembling in the cell lysates (Fig 5C) were much higher than that used in living cells (Fig 5B), in which the drugs at lower dosages operated cumulatively over long incubation times. Thus, lysing the cells at 25°C for 10 min with the amounts of 3MCIC- or paclitaxel used in living cells provided the same P/S-tubulin ratio as vehicle control (data not shown), indicating that the effects in Fig 5B occurred completely in living cells, not during the lysis-incubation period.

### Changes of cell-cycle control proteins upon 3MCIC treatment

**Fig 6:**
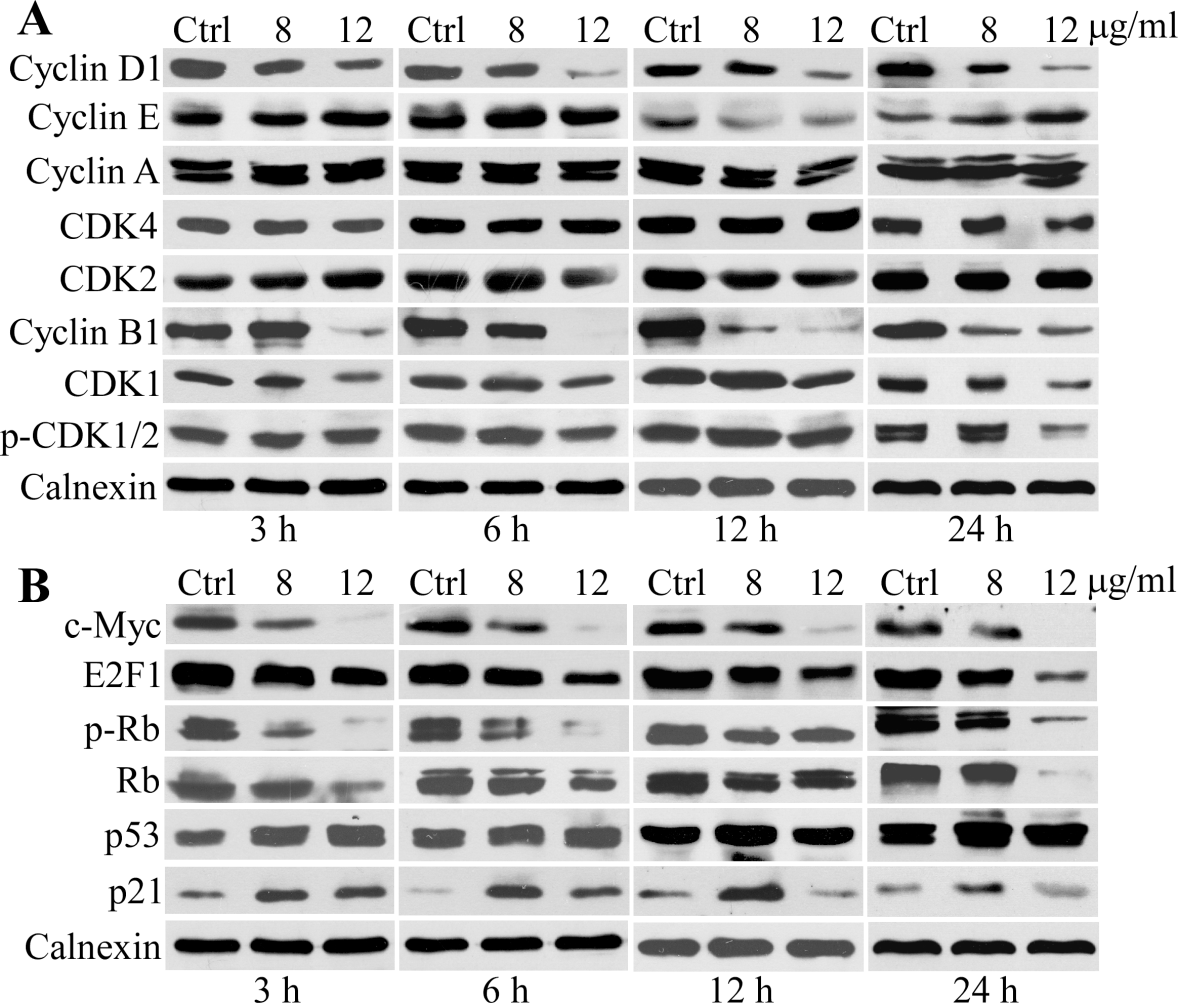
Changes of cell-cycle control proteins in 3MCIC-treated HepG2 cells. Cells were treated with 8 or 12 μg/ml 3MCIC at indicated times respectively. Calnexin was used as loading control. **(A)** Western blotting of cyclins and CDKs. **(B)** Western blotting of c-Myc, E2F1, Ser567 phosphorylated p-Rb, Rb, p53 and p21.

3MCIC treatment caused drastic reduction of cyclin D1 and cyclin B1 in HepG2 cells, especially at higher dosage and prolonged incubation times (Fig 6A). However, cyclin A, CDK4 and CDK2 were not significantly affected. The fluctuation of cyclin E levels lacked a meaningful trend. 3MCIC at 12 μg/ml reduced CDK1 under all tested incubation times, but reduction of inactivated p-CDK1/2 (the Thr14Tyr15-phosphorylated form) was only observed after 24 h treatment. Consequently, the percentage of inactivated CDK1 increased significantly (data not shown). These results suggested that 3MCIC could reduce CDK1 protein levels while concomitantly inhibiting CDK1 activation. Moreover, 3MCIC downregulated c-Myc, E2F1, Ser567-phosphorylated p-Rb and total Rb; but upregulated p53 and p21 (Fig 6B).

### 3MCIC inhibits both GSK3β and the Wnt/β-catenin pathway

**Fig 7:**
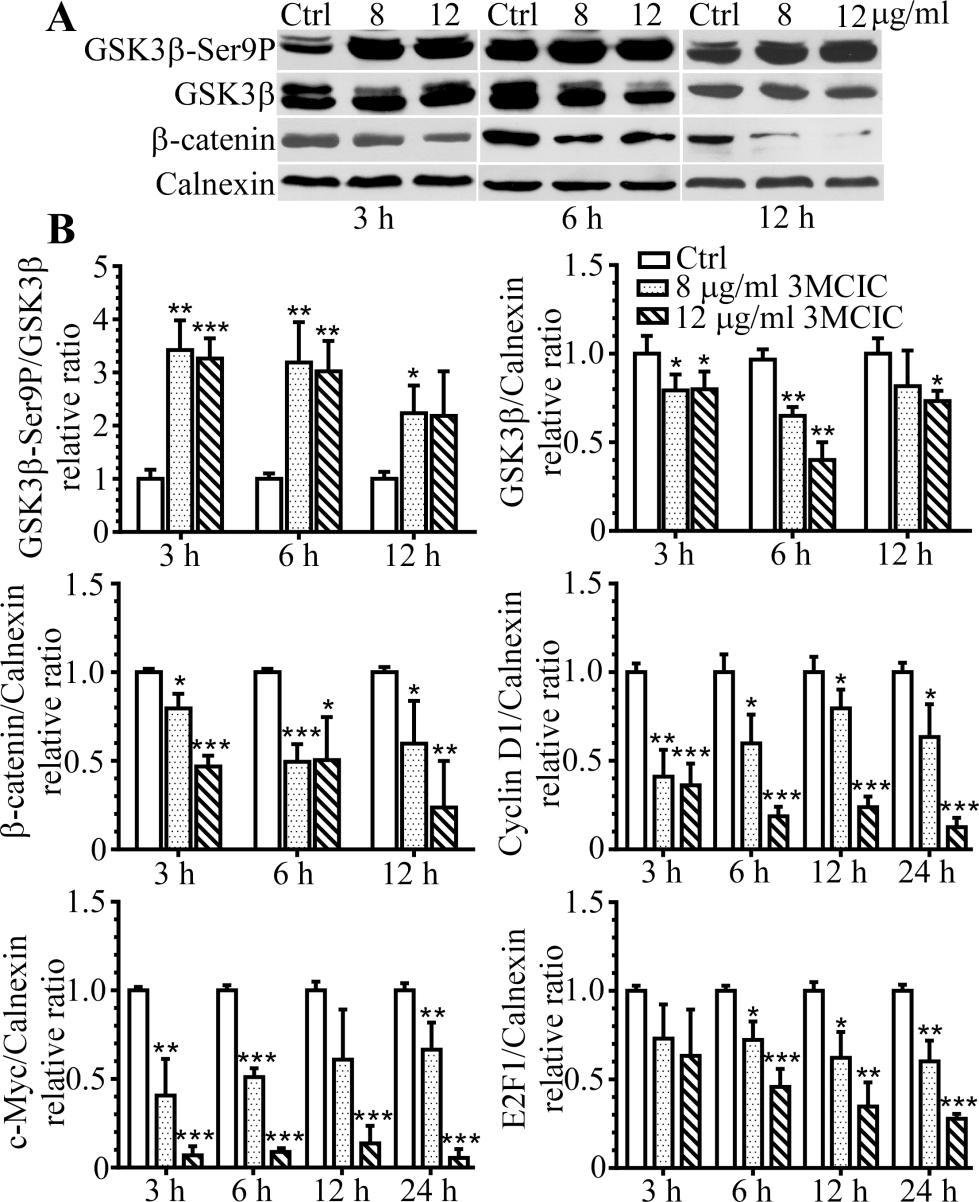
Concomitant inhibition of GSK3β and the Wnt/β-catenin pathway in 3MCIC-treated HepG2 cells. **(A)** Western blotting of GSK3β-Ser9, total GSK3β and β-catenin, respectively. **(B)** Quantification of 3MCIC-induced changes in the Wnt/β-catenin-GSK3β pathway. Normalized relative band-intensity ratios of pathway component/calnexin in 3MCIC-treated groups over controls were plotted. *P<0.05, **P<0.01 and ***P<0.001 vs control (n = 3).

3MCIC treatment increased the enzymatically inactivated form of GSK3β-Ser9P and reduced total GSK3β proteins. Therefore, the GSK3β-Ser9P/GSK3β ratio elevated significantly (Fig 7). These results are in accordance with the fact that Ser9 phosphorylation on GSK3β causes GSK3β inactivation and subsequent degradation through the ubiquitin-proteasome system [41]. Since GSK3β can regulate the Wnt/β-catenin pathway, we detected β-catenin and found it reduced drastically upon 3MCIC treatment. Moreover, the down-stream elements of the Wnt/β-catenin pathway, including cyclin D1, c-Myc and E2F1, were all significantly downregulated (Fig 7B, representative Western blot results were shown in Fig 6).

### Early and multiple cell-death pathway activations in 3MCIC-treated cells

**Fig 8:**
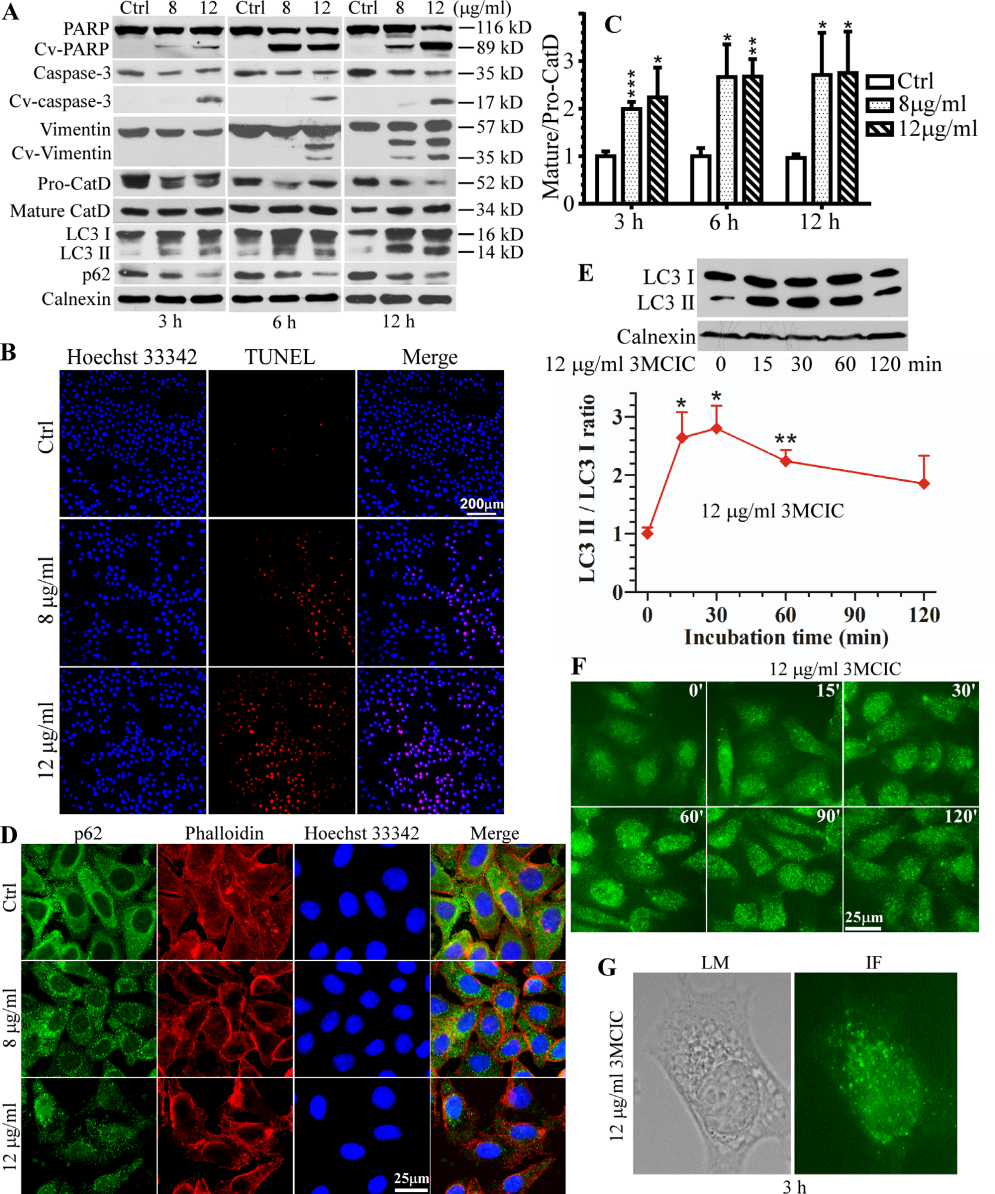
Activation of cell death pathways in 3MCIC-treated HepG2 cells. **(A)** Western blots showing 3MCIC-induced PARP, caspase-3 and vimentin cleavages, CatD maturation, LC3 I & II upregulation, and p62 downregulation. **(B)** The TUNEL assay after 3MCIC treatment for 12 h. **(C)** Normalized relative band-intensity ratios of mature/pro-CatD in 3MCIC-treated groups over controls were plotted. **(D)** Immunofluorescence of HepG2 cells treated with 3MCIC for 3 h, and stained with p62 mAb (green), phalloillin (red) and Hoechst 33342. **(E)** The early-stage alternations of LC3 under 12 μg/ml 3MCIC treatment. Normalized relative band-intensity ratios of LC3 II/LC3 I in 3MCIC-treated groups over controls were plotted. In **C** & **E**, *P<0.05, **P<0.01 and ***P<0.001 vs control (n = 3). **(F)** LC3 immunofluorescence of HepG2 cells treated with 12 μg/ml 3MCIC at the indicated times. **(G)** A HepG2 cell was observed either by a light microscope (LM) to show the vacuoles, or by immunofluorescence (IF) with a LC3 mAb, after incubation with 12 μg/ml 3MCIC for 3 h.

To clarify the mechanisms of cell death in 3MCIC-treated HepG2 cells, we examined the molecular signatures of apoptosis and autophagic cell death (Fig 8). PARP cleavage, evidenced by the 89 kD fragment or cleaved-PARP (Cv-PARP), occurred within 3 h when the cells were treated with either 8 or 12 μg/ml of 3MCIC (Fig 8A). Caspase-3 activation by enzymatic cleavage (Cv-caspase 3) was also evident at 3 h under treatment with 12 μg/ml 3MCIC. A key down-stream event following the caspase activation cascade is vimentin cleavage [41], which resulted in two fragments (Cv-vimentin). DNA fragmentation is the hallmark of apoptosis. We used TUNEL assay to detect DNA breaks in HepG2 cells treated with 3MCIC for 12 h. Numerous cells became TUNEL-positive under the influence of 3MCIC, and the number of positive cells increased using higher dosage of 3MCIC (Fig 8B). These data suggest that 3MCIC rapidly activate the apoptotic pathway, especially under higher dosage.

Lysosomal membrane permeabilization results in activation and release of cathepsin D (CatD), which is usually considered as a necrosis marker. However, CatD has multiple essential roles in lysosome-mediated cell death that is correlated with either necrosis or apoptosis [46]. In 3MCIC-treated cells, the non-activated 52 kD pro-CatD decreased, whereas the mature and enzymatically active 34 kD CatD increased (Fig 8A). Consequently, the mature/pro-CatD ratios significantly elevated at all the doses and incubation times we tested (Fig 8C), suggesting that lysosome instability is an early and important event in 3MCIC-induced cell death.

Vacuole accumulation and fusion were characteristically observed in 3MCIC-treated cells (Fig 3). In accordance with pro-CatD cleavage and activation, it was likely that the vacuoles were originated from endosomes and lysosomes. However, autophagic cell death is also characterized by vacuole accumulation. Therefore we analyzed the autophagy markers LC3 and p62 [47] in 3MCIC-treated cells, and found that both LC3 II accumulation and p62 consumption were evident within 3 h of treatment (Fig 8A). In autophagy, p62 is recruited onto the surfaces of damaged organelles and denatured protein aggregates. Recruited p62 labels autophagic foci and initiates the activation of the autophagic machinery. Consequently p62 is wrapped and digested in autophagosomes, causing its depletion [47]. In control cells (Fig 8D), an intense and diffuse immunofluorescence pattern for p62 was observed throughout the cytoplasm, indicative of resting p62 pools. However, a small fraction of p62 was localized in tiny granules, which reflected the basal levels of autophagic foci. Contrarily, most of the p62 signals were localized in granules in cells treated with 8 μg/ml of 3MCIC for 3 h, and the overall fluorescence intensity of p62 was drastically reduced under 12 μg/ml of 3MCIC treatment.

Transformation of LC3 I into activated LC3 II is another important signature of autophagy. It was reported that autophagic regulation of LC3 has two distinct stages [48]: When cells are challenged with autophagic stimuli, LC3 II accumulates within 30 min whereas LC3 I is depleted. However, at the later stage, prolonged activation of autophagy results in intensive expression of LC3-mRNA, causing significant elevation of both LC3 I and LC3 II protein levels. Such a two-stage scenario was clearly observed in 3MCIC-treated cells. Both LC3 I and II accumulated within 3 to 12 h of 3MCIC treatment, which reflected the later stage (Fig 8A). We further analyzed the early stage of LC3 modification by Western blotting (Fig 8E & 8F), and revealed a remarkable elevation of LC3 II within 15 min. Quantification of the LC3 bands showed that the normalized LC3 II/LC3 I ratio elevated significantly at 15 min and reached a peak at 30 min, but decreased after prolonged treatment. Consistent with Western blotting, LC3 immunofluorescence in the 15 to 120 min time-windows showed that the punched and granular LC3 foci were most intensive at 30 to 60 min (Fig 8F). The images of a representative cell after 3 h treatment were displayed (Fig 8G), in which the LC3 foci were co-localized with the vacuoles seen in the phase-contrast field. Taken together, our data suggest that 3MCIC can induce intensive autophagy in HepG2 cells.

### Target identification by LAC-MS and Western blotting

**Fig 9:**
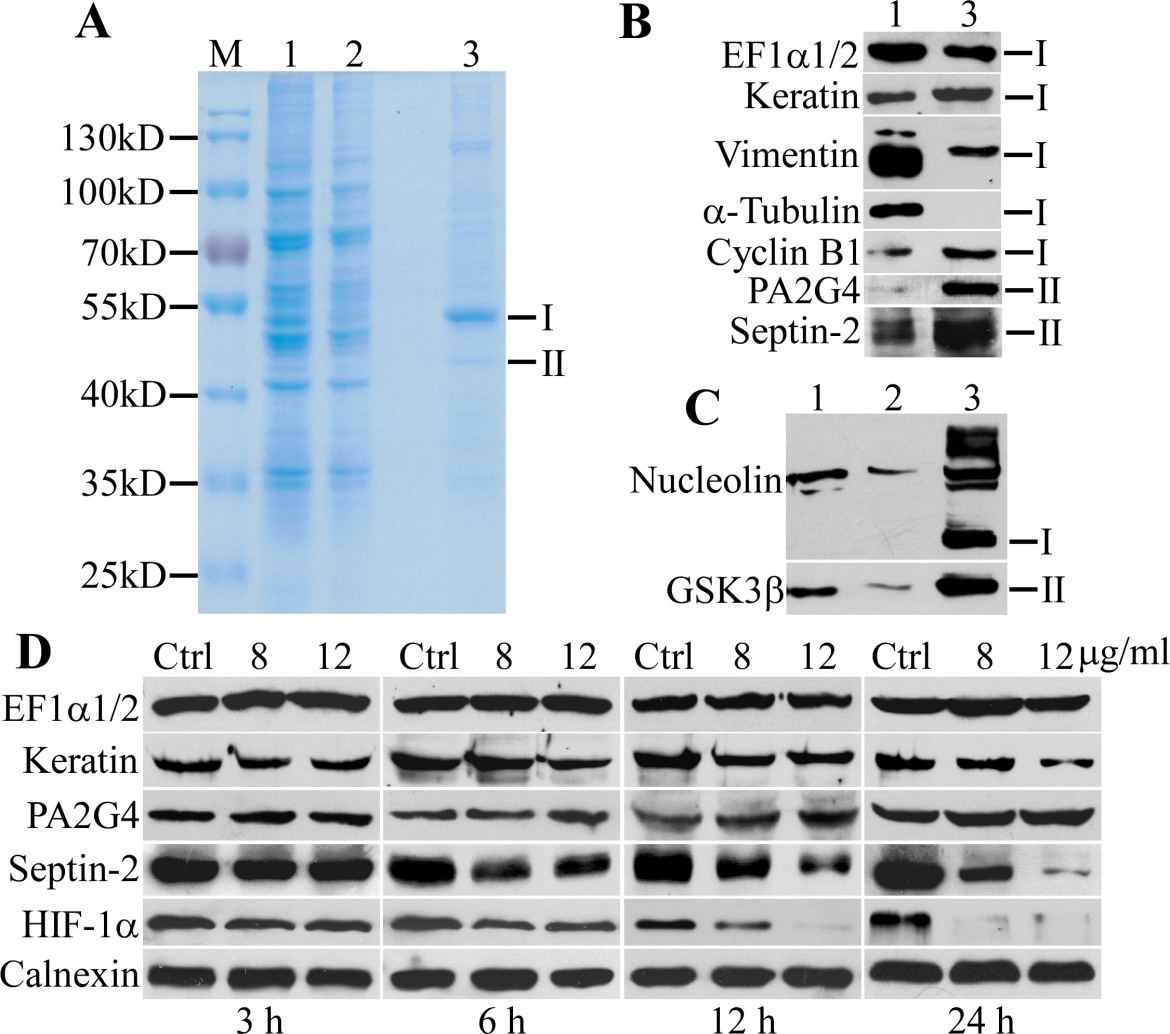
Target isolation by LAC and changes of target proteins in 3MCIC-treated cells. HepG2 cell lysates were loaded onto the LAC column, and the eluted proteins were analyzed by SDS-PAGE **(A)** and Western blotting **(B & C)**. The positions of band I and II are indicated at the right side. M is molecular weight markers. Lane 1: HepG2 cell lysates (20 μg total protein loading). Lane 2: a flow-through fraction from the column (15 μg for **A** and 20 μg for **C**). Lane 3: the column eluent (5 μg). **(D)** Regulation of target proteins in 3MCIC-treated HepG2 cells.

To clarify the molecular targets of 3MCIC, we prepared a LAC column using the Sephacryl S1000-C_12_-3MCIC resins (S1 Fig). Cultured HepG2 cells were collected and lysed with a low salt buffer. The LAC column was loaded with the cell lysates, washed, and then eluted with a high salt buffer. Proteins eluted from the LAC column were analyzed by SDS-PAGE and revealed a major band (band I) about 51 kD and a minor band (band II) about 44 kD (Fig 9A). The two bands were cut, digested and analyzed with MS. Notably, both band I and band II contained multiple proteins (S1 Excel File). Abundant proteins identified in the two bands could be grouped into four major functional categories:

1) Cytoskeleton proteins, including septin-2, septin-6, septin-7, septin-10, septin-11, microtubule-associated protein 4 (MAP4), vimentin, keratins, etc. 2) Proteins for ribosome assembly and mRNA translation, including elongation factors EF1α1 (EF1A1), EF1α2 (EF1A2), EF1γ, 60S ribosomal protein L3 & L4 (RPL3, RPL4), nucleolin, eukaryotic translation initiation factor 2 subunit 3 (EIF2S3), eukaryotic translation termination factor 1 (ETF1), etc. 3) Cell cycle and signaling proteins, including cyclin B, GSK3β, proliferation-associated protein 2G4 (PA2G4 or EBP1), vasodilator-stimulated phosphoprotein (VSGP), integrin-linked protein kinase (ILK), etc. 4) Vesicular transport regulators, including isoform 2 of AP-2 complex subunit mu (AP2M1), isoform 2 of Arf-GAP with SH3 domain (ASAP2), etc.

To further confirm the LAC-MS data, we analyzed the LAC-eluted fractions by Western blotting using antibodies specific to EF1α1/2, nucleolin, septin-2, vimentin, pan-cytokeratin, EBP1/PA2G4, cyclin B1 and GSK3β; and all these proteins were identified in the column eluents (Fig 9B & 9C). Importantly, septin-2, cyclin B1, EBP1/PA2G4, GSK3β and nucleolin (with several isoforms) were highly enriched in the column eluents, in spite of a much lower total protein loading for the LAC eluents than that for the cell lysates in Western blotting. However, 3MCIC did not bind to tubulin directly, since no tubulin band was detected in the LAC eluents (Fig 9B). Binding of cyclin B to the 3MCIC-resin was highly specific; since cyclin D, cyclin E, cyclin A and CDKs were all absent in the LAC eluents (data not shown).

In 3MCIC-treated HepG2 cells, cyclin B1 levels were downregulated (Fig 6A). Similarly, GSK3β, another 3MCIC target, was also downregulated (Fig 7). To reveal the changes of other 3MCIC targets, we analyzed some of the 3MCIC-targeted proteins in 3MCIC-treated HepG2 cells (Fig 9D). Septin-2, which bound to the 3MCIC-resin, was downregulated drastically. The pan-cytokeratin levels decreased moderately under higher 3MCIC dosage and prolonged treatment. Nevertheless, such a decrease might result from caspase-dependent degradation, in a way similar to vimentin (Fig 8A). Whether binding of 3MCIC to vimentin and keratins has a direct destabilizing effect on these intermediate filament components remains further study. On the other hand, though EF1α1/2 and EBP1/PA2G4 bound to 3MCIC, their levels were not changed, indicating that ligand influence on target stability is complex [49]. Interestingly, though HIF-1α was not detected in the LAC eluents, it was downregulated by 3MCIC (Fig 9D).

## Discussion

### 3MCIC is a unique cytoskeleton-targeting compound

3MCIC was designed as a multitarget anticancer lead that structurally combined the 3,4,5-trimethoxyphenyl, chalcone and isatinyl moieties. Cytotoxicity assays on cultured HepG2 cells revealed that 3MCIC was more potent than sorafenib. Sorafenib is a multitarget inhibitor that can directly inhibit dozens of essential kinases in cell proliferation and angiogenesis pathways [22]. However, lack of powerful disturbance on the cytoskeleton in cancer cells is most likely the major limit for sorafenib's activity. Contrarily, our studies revealed that 3MCIC not only inhibited multiple essential components in the cell proliferation machinery, but also drastically altered the cytoskeleton on multiple dimensions.

Target identification is the bottleneck of anticancer research and drug development [50,51]. Using 3MCIC-specific LAC-MS, we identified several interesting target groups, including the cytoskeleton and the translation machinery proteins. Cytoskeleton proteins such as septins, keratins, vimentin and MAP4 were abundant in the LAC eluents. Septins are a newly identified fourth class of cytoskeleton proteins that have a variety of physiological and pathological roles [52,53]. Aberrant expression of septins is involved in tumorigenesis, e.g., overexpression of septin-2,-8,-9,-11 and downregulation of septin-4 and septin-10 have been identified in a wide variety of cancer types [54]. Thus, finding novel molecules targeting septins are highly desired in anticancer research. To our knowledge, forchlorfenuron (FCF) is the only reported septin-binding small molecule, and its anticancer effects have been evaluated [55,56]. FCF binds to septin-9_i1 and inhibits HIF-1α, implying that septin inhibitors may have anti-angiogenesis activity [56]. In accordance with the report, we also showed that 3MCIC could drastically downregulate HIF-1α protein levels (Fig 9D). Unlike simultaneous downregulation of its direct-target septin-2 and indirect-target HIF-1α by 3MCIC, however, FCF dose not alter the expression levels of its direct-target septin-9_i1 [56]. Thus, our findings show that 3MCIC is another septin-binding compound that is both structurally and pharmacologically different from FCF.

Surprisingly, despite structural similarity of 3MCIC with CA-4, 3MCIC did not bind to tubulin. Moreover, 3MCIC increased the P/S-tubulin ratio both in cultured HepG2 cells and in the cell lysates, which was opposite to the MT-disruption effect of CA-4. We suggest that the MT-stabilizing effect of 3MCIC is related with its ability to bind and downregulate septins, since it has been reported that septin depletion by RNAi stabilizes MT dynamics through abolishing septin’s inhibition on the MT-stabilizing protein MAP4 [57]. Moreover, MAP4 can bind and recruit cyclin B1 to MTs [58], which may explain co-elution of MAP4 and cyclin B1 with septins from the 3MCIC-resins.

Importantly, in 3MCIC-treated HepG2 cells, MTs became entangled and aggregated, implying that intracellular trafficking along MTs was seriously hampered. A recent report revealed that both the MT-disrupting agent vincristine and the MT-stabilizing agent paclitaxel block trafficking of multiple DNA damage-repair proteins on MTs in the cytoplasm, and therefore prevent these proteins from entering the nucleus [59]. Such a mechanism explained both the cytotoxic activity of these agents at interphase and the synergistic combination of MT-targeting agents and DNA-damaging agents in anticancer regimens. Taken together, we suggest that 3MCIC’s cytotoxic activity can be at least partially attributed to interference with MT functions through targeting septins.

The rounding phenotype in 3MCIC-treated HepG2 cells is also related with cytoskeleton perturbations including downregulation of paxillin and entanglement of F-actin fibers. Paxillin is an essential scaffold protein for assembling of focal adhesion plaques and actin stress fibers. Recent findings reveal that septin-9 can stabilize focal adhesions and actin stress fibers, while septin-9 knockdown inhibits cell migration [60,61]. Based on these findings, we propose that by binding and inhibiting septins, 3MCIC can deplete paxillin and disrupt stress fibers, and consequently block cancer cell migration and metastasis. Nevertheless, further study along this line is needed.

The presence of vimentin and cytokeratins in the eluents of the 3MCIC-resins is also noteworthy, since both of these intermediate filament proteins are considered as anticancer therapeutic targets [62,63]. Vimentin overexpression is highly correlated with tumorigenesis of HCC [64], since it can promote cell adhesion and migration. Overexpressed keratins are a prominent signature of HCC and thus have diagnostic values [65]. However, small molecules acting as vimentin- or keratin-ligands were seldom reported. It is interesting to further investigate the binding nature of 3MCIC to vimentin and keratins.

### 3MCIC targets the translation machineries

Another prominent target group of 3MCIC involves proteins in the translation machineries, i.e., nucleolin, RPL3, RPL4 for ribosome assembly; and EF1α1/2, EF1γ, EIF2S3, ETF1 for mRNA translation. It is highly likely that by blocking the translation machineries, 3MCIC treatment initiates a cellular starvation signal, which results in autophagy within minutes. We believe that the target proteins within this group are very promising research objects, and among them necleolin and EF1α1/2 are especially important.

Nucleolin is a multi-functional protein that not only regulates ribosome biogenesis, but also participates in chromatin remodeling, cell-cycle signaling, cellular stress and DNA-damage responses, apoptosis inhibition, centrosome and spindle integrity maintenance, telomerase assembly, etc [66]. Consistent with its versatile roles, subcellular localization of nucleolin is diverse, notably including cell membranes. Importantly, nucleolin is an essential protein in the maintenance of embryonic stem cell identity and homeostasis against differentiation-inducing oxidative stress [67]. Nucleolin overexpression is highly correlated with cancer, including HCC [66,68]. Engineered anti-nucleolin antibody fragment targeting cell surface necleolin on breast cancer and HCC cells selectively killed the cancer cells and reduced tumor volumes in mouse models, without injuring the normal cells [69]. Besides, EF1α1/2, a pair of 3MCIC-binding proteins known as GTP-binding translation factors, are also pleiotropic proteins with multiple roles in oncogenesis of various cancers including HCC, and are therefore considered as novel anticancer targets [70].

It was reported recently that yeast septin interactors were predominantly nuclear proteins and proteins involved in ribosome biogenesis in yeast cells arrested in G1 phase by α-factor [71]. However, the significance of the findings in the context of mammalian systems remains to be investigated. Consistent with the report, LAC with 3MCIC-resins exhibited co-enrichment of septins with multiple proteins involved in ribosome biogenesis and translation. In reference to the aforementioned discussion about the effects of 3MCIC on the cytoskeleton components, it is highly likely that septins are the pivotal targets of 3MCIC.

### 3MCIC targets the cell-cycle control network and the Wnt/β-catenin-GSK3β pathway

Our data showed that 3MCIC affected the cell-cycle regulation network at multiple levels, including depletion of cell-cycle driver proteins and upregulation of cell-cycle inhibitory proteins. LAC-MS data uncovered that both cyclin B and GSK3β bound to the 3MCIC-resins. Moreover, both of the proteins were downregulated in 3MCIC-treated HepG2 cells. The Wnt/β-catenin-GSK3β pathway is a pivotal pathway to control the balance of cell proliferation and differentiation, and its malfunction is one of the most evident oncogenic signatures in HCC [13–16]. Being a master kinase that is located at the converging point of multiple signaling pathways, GSK3β can regulate the half-life of thousands of proteins that are involved in cell proliferation and death [72,73]. GSK3β has long been considered as an important anticancer target, since its inhibitors have shown promising therapeutic potentials for certain cancer types [74,75].

However, the roles of GSK3β in cancer are controversial, since it can act both as an oncogene product and as a tumor suppressor, depending on the context of the pathway circuits within a certain cell. In normal cells, GSK3β inactivation usually activates the Wnt/β-catenin pathway by increasing β-catenin levels, which promotes proliferation. Moreover, GSK3β inhibition upregulates HIF-1α by increasing the expression of nucleolin, which stabilizes HIF-1α mRNA [76]. Fortunately, although binding and downregulation of GSK3β by 3MCIC were observed, concomitant reduction of β-catenin, nucleolin and HIF-1α levels were also evident, implying that the harmful oncogenic effects of GSK3β inhibition were blocked by 3MCIC. Interestingly, Yang *et al*. reported that suppressing GSK3β activity in cells under starvation resulted in a Bif-1-dependent autophagy-induced necrosis, and that blocking autophagy switched the cells to apoptosis [77]. Accordingly, our data implied that extensive interference with the translation machinery and simultaneous blocking of GSK3β by 3MCIC activated multiple cell-death pathways, so that the autophagic, necrotic and apoptotic pathway signatures were all observed.

It has been reported that dozens of isatin derivatives are GSK3β inhibitors [33–35]. Thus, 3MCIC also preserves the GSK3β-inhibitor activity assigned to the isatinyl group. Moreover, our data showed that septins, nucleolin and GSK3β were all highly enriched in the LAC eluents, suggesting that among many competing targets, these three proteins bound more tightly and specifically to the 3MCIC-resins. Taken together, we propose that septins, nucleolin and GSK3β are the most important targets for 3MCIC to exert its cytotoxic effect toward cancer cells.

## Supporting Information

S1 Excel File Target proteins identified by LAC-MS. The file contains two sheets: the band I and the band II sheets list the 3MCIC-binding proteins identified by MS from the two corresponding bands in the SDS-PAGE gel (Fig 9A). More detailed message in the sheets can be seen by clicking the plus-sign buttons on the left column.
(XLS)

S1 Fig Scheme to prepare the LAC resins. Sephacryl S1000 resins (**3**) react with 1,12-dibromododecane in excess to obtain the activated resins (**4**), which react in the next step with excessive 3MCIC to obtain the Sephacryl S1000-C_12_-3MCIC resins (**5**). The inset shows that the original white Sephacryl S1000 resins (**3**) changed to orange color (**5**) when 3MCIC was covalently coupled to the resins.
(TIF)
