# Supplementary figures and images for: A Hybrid Chalcone Combining the Trimethoxyphenyl and Isatinyl Groups Targets Multiple Oncogenic Proteins and Pathways in Hepatocellular Carcinoma Cells
Source: PLoS One. 2016 Aug 15;11(8):e0161025. doi: 10.1371/journal.pone.0161025 (PMC4985065; doi:10.1371/journal.pone.0161025)

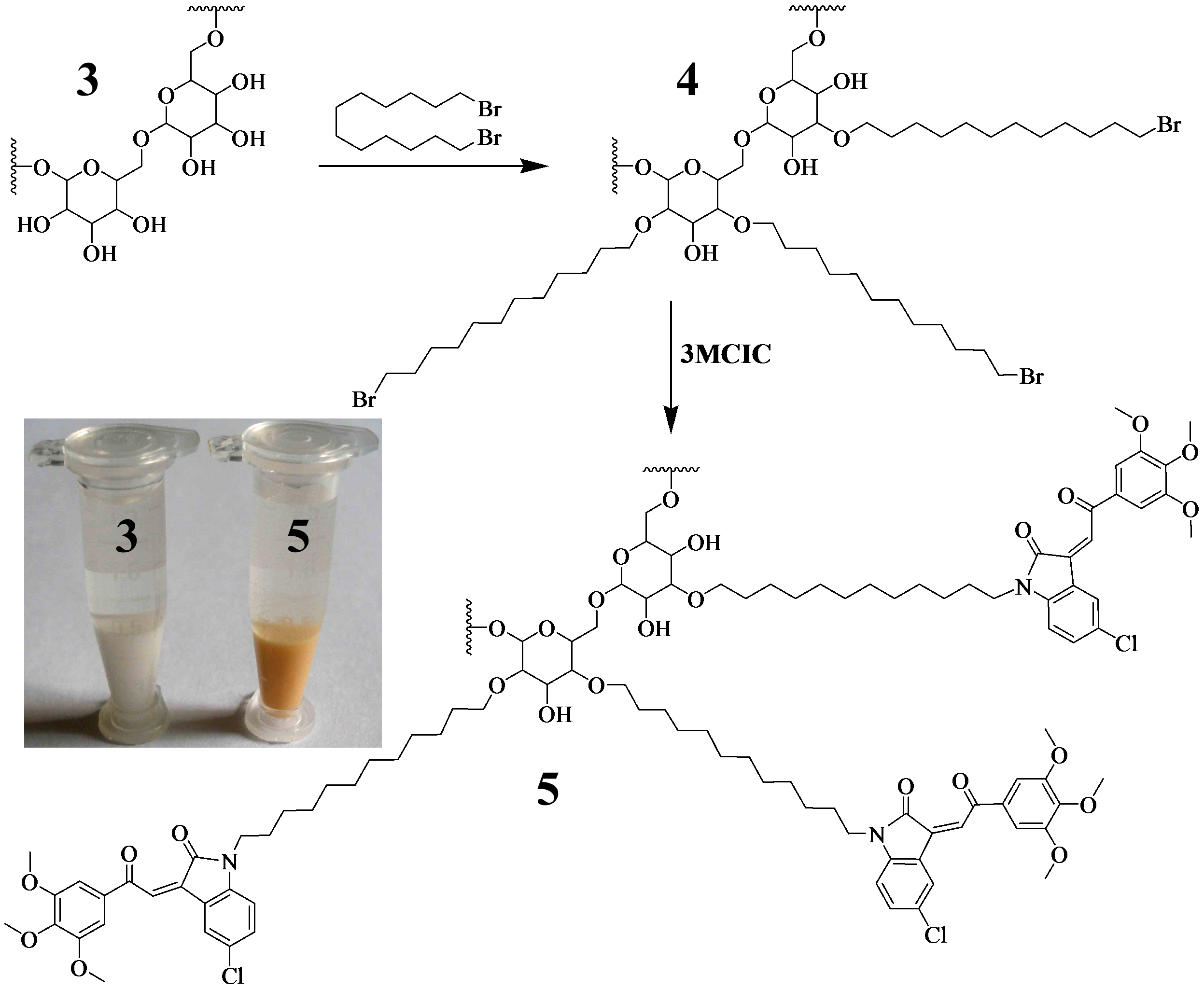

Supplement: S1 Fig — Sephacryl S1000 resins (3) react with 1,12-dibromododecane in excess to obtain the activated resins (4), which react in the next step with excessive 3MCIC to obtain the Sephacryl S1000-C12-3MCIC resins (5). The inset shows that the original white Sephacryl S1000 resins (3) changed to orange color (5) when 3MCIC was covalently coupled to the resins. (TIF) [file pone.0161025.s002.tif]
